# Cases of IIeus

**Published:** 1831-04-01

**Authors:** 


### LA CHARITE.

### CASES OF ILEUS. M. CORBIN.

ALTHOUGH ileus is most frequently dependent on a mechanical cause, it sometimes takes place without any appreciable change of structure in the intestinal tube. The difficulty is to distinguish between the one kind and the other; since we cannot but suppose that the treatment should vary according to the cause of the disease. On this account we deem every fact that can be ascertained relative to ileus, as adding to the sum total of useful knowledge.

*Case.* 1. Francis Jallat, aged 25 years, entered La Charité on the 15th December, 1828, under M. Lerminier. He had coughed for some time, and complained of nocturnal perspirations, diarrhœa, and progressive emaciation. Percussion and auscultation detected no disease in the chest. The abdomen was neither tender nor tense. Such continued his condition on the 1st of January, 1829, when the reporter examined him, and for some days afterwards. On the 10th of January, the abdomen became tender, tense, and swelled, with violent colicky pains, vomiting, small quick pulse, shrunk features, &c. He had had no motion for 36 hours. Fomentations and lavements produced several copious evacuations, with relief of all the symptoms, and a restoration to his ordinary condition. In the night of the 21st of January, he was seized with a similar attack, and a dozen of leeches were applied to the anus, while emollient lavements were thrown up, without inducing stools. Oil, syrup, and laudanum were then administered internally, with the effect of evacuating the bowels. An interval of six weeks took place before a third attack. On the 6th of March, he had colic, vomiting, distended abdomen; and again the oily opiates dispersed the symptoms. On the 18th of the above month another invasion of the malady; but the remedies no longer afforded relief, and he sunk on the

25th of March, under porraceous vomiting, dreadful pains in the abdomen, and unconquerable constipation.

*Dissection.* There was a triple constriction (rétrécissement) at the lower portion of the ileum—purulent effusion into the peritoneal cavity—adhesion of several portions of the intestines, the surface of which was much injected with blood, and of a dark colour. These adhesions, of which a minute but rather confused description is given, entangled two or three folds of small intestine, and bound them down so as to produce complete internal herniæ. On opening the small intestines, there was found, near the termination of the ileum, a valvular and mechanical obstruction, the description of which is also rather obscure. Above this obstruction, there was considerable dilatation of the gut—nearly equal to the size of the stomach. In this part the parietes of the intestine were greatly thickened. These wounds, at some distance from each other, two other contractions and obstructions of the gut, but not so complete as the first.

*Case* 2. Catharine Lominet, 16 years of age, of feeble constitution, entered the hospital on the 2d of September, 1829, under M. Chomel. She had been ill about six months, with colicky pains, diarrhœa, flatulence, heart-burn, nausea, and even occasional vomitings, especially during the few days preceding her entrance into hospital. On examining the abdomen a good deal of gurgling noise and borborygmi were heard. In the hollow of the right ilium, a tumour was felt, the size of an egg, which M. Chomel considered as an enlarged ovarium. There was no hernia on either side. Lavements and some inert remedies were prescribed. For some days the belly was distended, and few or no stools were passed. On the 7th September, the colicky pains were more than usually violent, and some porraceous matters were thrown up by vomiting, of a suspicious smell, but not decidedly stercoraceous. Constipation still continued obstinate.—There was evidently some strangulation of intestine in existence. The cause or seat was naturally referred to the right iliac region. Various means were used, especially lavements, ptisans, and ice to the abdomen; but no leeches were applied. Scammony, in considerable doses, was next administered, and some trifling evacuations were procured from the bowels. By these she was partially relieved. Towards the 18th of September a diarrhœa came on, and the doses of scammony were diminished or suspended. The tumour in the iliac fossa remained, and she was discharged from the hospital, in a poor state of health, on the 8th of March, 1830. She had still some diarrhœa, and could not do without the use of occasional purgatives. She was seen six months after her discharge. The tumour remained, the bowels were irregular, and required cathartics.

*Case* 3. Madame Chassereau, aged 45 years, entered LA CHARITÉ, on the 7th November, 1829, under M. Chomel. For three years past she had been subject to constipation of the bowels, sometimes going a week without any evacuation. She occasionally experienced in the region of the cæcum, spreading thence through the abdomen in the shape of colic, with disposition to vomit, and much malaise, and restlessness. For five days before she entered the hospital, she had experienced violent pains in the abdomen, especially in the region of the cæcum, with intervals of ease, but with frequent inclination to vomit. There was neither tension nor tumescence of the belly; but pressure over the site of the caput coli could not be borne. The tongue was moist and white—the pulse rather quick—the skin hot and dry. Twenty leeches to the seat of pain, followed by an emollient cataplasm, and lavements. Next day she had two stools, and was much relieved. For three days the patient continued getting better; when, on the 12th November, there was a relapse, and on the 13th, the patient was considered to be in a very dangerous state. She had vomited a large bason full of unequivocally stercoraceous matters mixed with bile and other ingredients. She had hiccup, small pulse, and her voice was almost extinct. Her countenance was hippocratic. Seltzer water—lavements—fomentations. The sickness continued, and five basonfuls of matter were ejected, similar to those above-mentioned. The abdomen was covered with ice—cold injections were thrown up—and saline aperients were prescribed. The abdomen continued tense and painful. Forty-eight grains of scammony in pills were given. This medicine was followed by several evacuations from the bowels, and considerable relief. She was soon convalescent, and left the hospital in a few days cured.—ARCHIVES.

We have seen several cases where stercoraceous vomiting and other bad symptoms were present, and yet where recovery took place. On the other hand, we have seen patients die where large quantities of water could be injected into the bowels without difficulty, and where it was quickly vomited up, shewing that there was no permanent or organic obstruction in the bowels—at least in one direction. A case of this kind lately was attended by Mr. Blood, of Mount Street, and the writer, where a fine young woman was carried off in six or seven days by ileus, and where no previous disease had existed. Six or seven pints of warm water could be injected into the bowels, and soon afterwards it would be ejected by the mouth. There was neither tension nor swelling of the abdomen, and very little tenderness. The fever was trifling, and the tongue moist and clean almost till the last. No motions could be procured by the anus; but stercoraceous matters were ejected by the mouth. Unfortunately we could not obtain permission to examine the body.

